# P-505. Integrating PrEP Services into a Gynecology Clinic in Alabama: A Qualitative Analysis of Key Determinants

**DOI:** 10.1093/ofid/ofae631.704

**Published:** 2025-01-29

**Authors:** Hannah Goymer, Madeline Pratt, Kaylee W Burgan, Desiree Phillips, Lynn T Matthews, Mirjam-Colette Kempf, Bernadette Johnson, Michael J Mugavero, Audra Williams, Latesha Elopre

**Affiliations:** University of Alabama at Birmingham Marnix E. Heersink School of Medicine, Birmingham, Alabama; University of Alabama at Birmingham, Philadelphia, Pennsylvania; University of Alabama at Birmingham (UAB), Birmingham, Alabama; University of Alabama at Birmingham, Philadelphia, Pennsylvania; University of Alabama at Birmingham Heersink School of Medicine, Birmingham, Alabama; University of Alabama at Birmingham, Philadelphia, Pennsylvania; UAB, Birmingham, Alabama; UAB, Birmingham, Alabama; University of Alabama Birmingham, Birmingham, Alabama; University of Alabama of Birmingham, Birmingham, AL

## Abstract

**Background:**

In the Southeastern United States, Black women are disproportionately diagnosed with HIV compared to White women. Client-centered approaches are needed to increase uptake of HIV prevention tools, including pre-exposure prophylaxis (PrEP). Gynecology (GYN) clinics are uniquely positioned to provide PrEP for women and Black women prefer receiving PrEP from GYN providers, but PrEP delivery in GYN settings has been limited. Thus, we evaluated barriers and facilitators to PrEP provision in GYN clinics.

**Figure:**
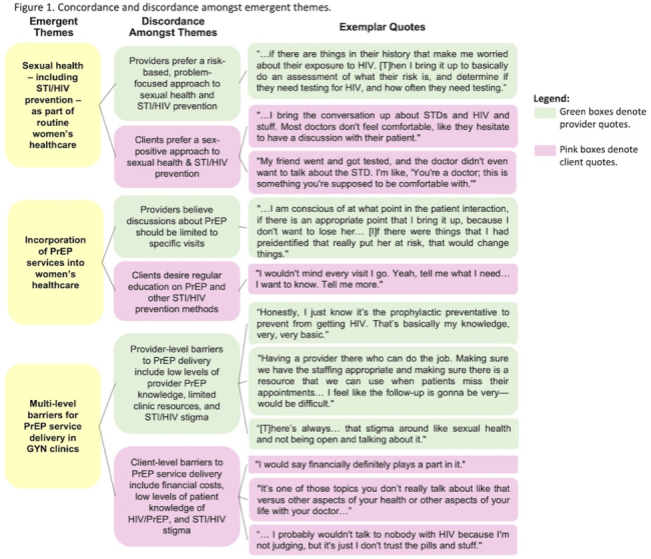

**Methods:**

From 08/2022-04/2023, we conducted dyadic focus groups and in-depth interviews with providers (i.e., physicians, nurses, and medical assistants) and clients accessing care at a university-affiliated GYN clinic in Alabama where clients face high STI burden. Interview guides grounded in the Information-Motivation-Behavioral Skills Model explored barriers to PrEP service delivery. Inductive qualitative analysis identified emergent themes from transcripts.

**Results:**

10 providers and 21 clients (100% Black, 100% female, median age 32 (range 19-44), 90.5% insured (67% with Medicaid)) were interviewed. Emergent themes included: (1) *Sexual health – including STI/HIV prevention – as part of routine women’s healthcare*. Providers preferred a risk-based approach to sexual healthcare while clients preferred a sex-positive approach. (2) I*ncorporation of PrEP services into women’s healthcare*. Both clients and providers supported that PrEP is important for women’s health. Providers felt PrEP discussions should be limited to specific visits whereas clients desired regular discussions of PrEP. (3) *Multi-level barriers for PrEP service delivery in GYN clinics*. Barriers included limited client and provider PrEP knowledge, inadequate clinic resources, financial costs, and STI/HIV stigma.

**Conclusion:**

Incorporating PrEP services into GYN clinics could increase PrEP uptake amongst Black women. Effective multi-level interventions should focus on increasing client and provider PrEP knowledge, integrating sexual health history assessment into routine care, mitigating STI/HIV stigma, and addressing structural barriers.

**Disclosures:**

**Latesha Elopre, MD**, Merck: Grant/Research Support

